# Evolution of *trappin *genes in mammals

**DOI:** 10.1186/1471-2148-10-31

**Published:** 2010-01-29

**Authors:** Akira Kato, Alejandro P Rooney, Yutaka Furutani, Shigehisa Hirose

**Affiliations:** 1 Department of Biological Sciences, Tokyo Institute of Technology, Yokohama, Japan; 2 United States Department of Agriculture, National Center for Agricultural Research Utilization, Microbial Genomics Research Unit, USA; 3 Current address: Laboratory for Neurobiology of Synapse, RIKEN Brain Science Institute, Saitama, Japan

## Abstract

**Background:**

Trappin is a multifunctional host-defense peptide that has antiproteolytic, antiinflammatory, and antimicrobial activities. The numbers and compositions of *trappin *paralogs vary among mammalian species: human and sheep have a single *trappin-2 *gene; mouse and rat have no *trappin *gene; pig and cow have multiple *trappin *genes; and guinea pig has a *trappin *gene and two other derivativegenes. Independent duplications of *trappin *genes in pig and cow were observed recently after the species were separated. To determine whether these *trappin *gene duplications are restricted only to certain mammalian lineages, we analyzed recently-developed genome databases for the presence of duplicate *trappin *genes.

**Results:**

The database analyses revealed that: 1) duplicated *trappin *multigenes were found recently in the nine-banded armadillo; 2) duplicated two *trappin *genes had been found in the Afrotherian species (elephant, tenrec, and hyrax) since ancient days; 3) a single *trappin-2 *gene was found in various eutherians species; and 4) no typical *trappin *gene has been found in chicken, zebra finch, and opossum. Bayesian analysis estimated the date of the duplication of *trappin *genes in the Afrotheria, guinea pig, armadillo, cow, and pig to be 244, 35, 11, 13, and 3 million-years ago, respectively. The coding regions of *trappin *multigenes of almadillo, bovine, and pig evolved much faster than the noncoding exons, introns, and the flanking regions, showing that these genes have undergone accelerated evolution, and positive Darwinian selection was observed in pig-specific *trappin *paralogs.

**Conclusion:**

These results suggest that trappin is an eutherian-specific molecule and eutherian genomes have the potential to form *trappin *multigenes.

## Background

Trappins are a family of small secretory proteins that possess an N-terminal transglutaminase-substrate (TGS) domain and a C-terminal whey acidic protein (WAP) domain [1]. The TGS domain consists of repeats of six semi-conserved amino acids, KGQDPV, that act as anchoring regions. In this case, the lysine or glutamine residues of these regions are cross-linked with extracellular-matrix proteins by the action of transglutaminases, which helps trappin molecules to become concentrated at the site of action [2-4]. In contrast, the WAP domain is a four-disulfide core region and is defined by eight conserved cysteine residues. The WAP domain of trappin shows anti-proteolytic [4-6] and antimicrobial [7-9] activities that allow it to act as an innate immune defense molecule. In fact, trappin-2 displays antibacterial activities against Gram-positive and Gram-negative bacteria [7-9]; it also has antifungal activity [9], and the antimicrobial activity is independent of its antiprotease function [9]. The most well characterized trappin is human trappin-2, which is also known as elafin, skin-derived antileukoproteinase (SKALP), elastase-specific inhibitor (ESI), or protease inhibitor 3 (PI3) [1,10]. It has strong inhibitory activity against leukocyte and pancreatic elastases and proteinase 3 [4-6], and shows anti-inflammatory activity [11] as well. The antiproteolytic and antimicrobial activities of trappin-2 are quite similar to those of secretory leukocyte protease inhibitor (SLPI) [12,13], which consists of two WAP domains with the second WAP domain being highly homologous to the WAP domain of trappin-2. Trappin-2 is expressed in the trachea, lung, gut, epidermis, esophagus, vagina, and oral epithelia [2,4]. In these tissues, the expression is induced by proinflammatory cytokines, such as interleukin-1 (IL-1) and tumor necrosis factor (TNF)-α [14,15].

The number of *trappin *genes varies among mammalian species. For example, humans and sheep have a single *trappin-2 *gene [16,17], while pigs have at least six: *trappin-1*, *trappin-2*, *trappin-3*, *trappin-7*, *trappin-8*, and *trappin-9 *[18,19]. At the other extreme are the mouse and rat, which lack *trappin *genes entirely [20], though the guinea pig has genes for trappin-12 and its derivatives caltrin II and seminal vesicle secretory protein (SVP), which lack TGS- and WAP-coding regions, respectively [21,22]. Despite the variance in copy number between the different mammalian lineages, all *trappin *genes are encoded by three exons. Exon 1 encodes a signal peptide, exon 2 codes for a TGS- and WAP-domains, and exon 3 encodes a 3' untranslated region [18,19,23]. While the exonic organization is highly conserved among various mammalian lineages, there is variation in the number of six-amino-acid repeats in the TGS domain [18,19]. Due to a point mutation of splicing site, guinea pig *trappin-12 *exceptionally lacks intron 2, which is present at the 3' noncoding region of the *trappin *gene [22]. A short interspersed element (SINE) is found in intron 2 of the *trappin *genes of the pig, wart hog, and collared peccary [18,19].

While we have mentioned several species that possess multiple *trappin *genes, it is not known if (1) these are exceptional cases or (2) *trappin *genes normally exist as a multigene family. In an attempt to find the answers, we analyzed genome databases developed by the Mammalian Genome Project http://www.broad.mit.edu/mammals/ and identified six *trappin *genes from the nine-banded armadillo (*Dasypus novemcinctus*) genome. The nine-banded armadillo belongs to the taxonomic order *Xenarthra*. Because this lineage is believed to be one of the most ancient lineages of placental mammals [24], the analyses of armadillo *trappin *genes are quite interesting because the duplication and evolution of armadillo *trappin *genes are expected to have occurred independently from other species. In contrast, we identified a single *trappin-2 *gene from the genome databases of many species including the chimpanzee, rhesus macaque, bushbaby, dog, cat, horse, cow, European shrew, European hedgehog, megabat, and microbat. This fact suggests that *trappin-2 *is the ancestral form of *trappin *genes, and *trappin*-null species such as mouse and rat are exceptional. Finally, we identified anciently duplicated *trappin-18 *gene in Afrotheria such as the elephant (*Loxodonta africana*), tenrec (*Echinops telfairi*), and hyrax (*Procavia capensis*), and *trappin*-related genes in chicken and opossum, suggesting that the gene family originated as far back as more than 100 million years ago.

## Results

### Identification of *trappin*, *SLPI*, and *trappin*-related genes in eutherian mammals, opossum, platypus, chicken, and zebra finch

**Figure 1:**
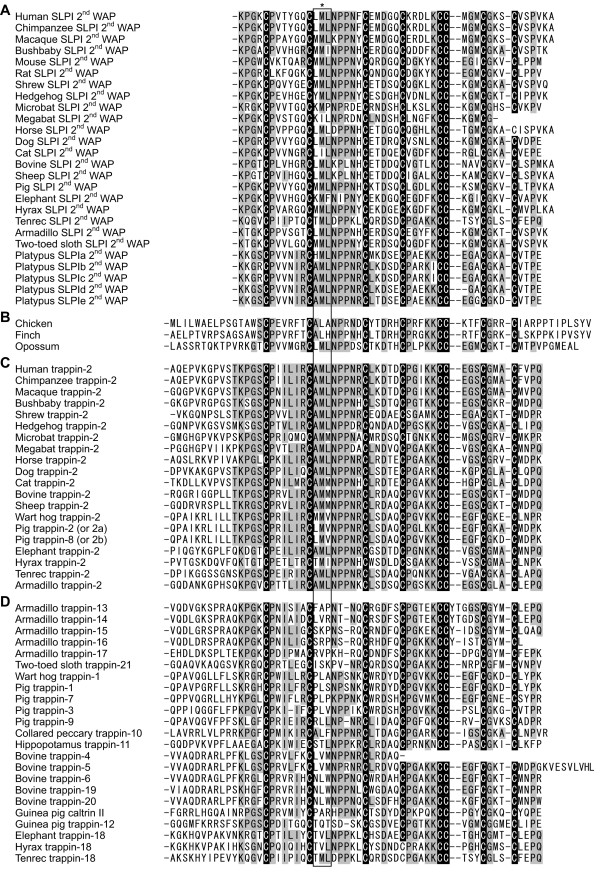
**WAP domain of the *trappin *gene family**. WAP domains of SLPI (A), *trappin*-related genes (B), trappin-2 (C), and the other species-specific trappin paralogs (D) are shown. Conserved and semiconserved residues among trappin-2 genes are indicated by light gray. Eight conserved Cys residues constituting the WAP motif signature sequence are shaded in black. The asterisk indicates the catalytically important Met residue. The variable region, which is thought to determine the specificity of WAP motifs, is boxed.

To estimate the origin of *trappin *genes, we analyzed the genome databases of eutherian mammals, opossum, platypus, birds (chicken and zebra finch), *Xenopus*, fish (*Danio rerio*, *Takifugu rubripes*, *Tetraodon nigroviridis*, *Gasterosteus aculeatus*, and *Oryzias latipes*), sea squirts (*Ciona intestinalis *and *Ciona savignyi*), insects (*Drosophila melanogaster*, *Anopheles gambiae*, and *Aedes aegypti*), and *Caenorhabditis elegans*. Typical *trappin *genes were identified only in the mammalian species (Figure 1C-D). A single homologous gene was identified in chicken, zebra finch, and opossum, and multiple homologous genes were identified in platypus, (Figure 1A-B). The other species did not show any homologous genes to *trappins *except for other WAP-coding genes with a low homology (data not shown).

The presence of the *SLPI *gene was also analyzed using the genome databases, and a single orthologous gene was identified in all mammalian species except for the guinea pig and the rabbit (Figure 1A). In contrast, there are no clear direct orthologs for the *SLPI *gene in the genome databases of chicken, zebra finch, and opossum, and the *trappin*-homologous genes are also the most homologous to *SLPI*. In platypus, the above-mentioned *trappin*-homologous genes encode two-WAP-domain proteins and may be the paralogs of the mammalian *SLPI *gene.

The *trappin*-related genes in chicken, zebra finch, and opossum have a single WAP-coding region but lack a TGS-coding region. Only the WAP-coding region is similar to *trappin *and the *SLPI *genes, but the other flanking regions lack any significant similarity except for a weak similarity in the signal-peptide coding regions (data not shown). The deduced amino acid sequence of the WAP domains of the *trappin*-related genes are shown in alignment with those of mammalian *trappin *and the *SLPI *genes (Figure 1B). The catalytically important Met residue (an asterisk in Figure 1, A and 1C) is conserved in the opossum and platypus genes but not in the chicken and zebra finch genes.

**Figure 2:**
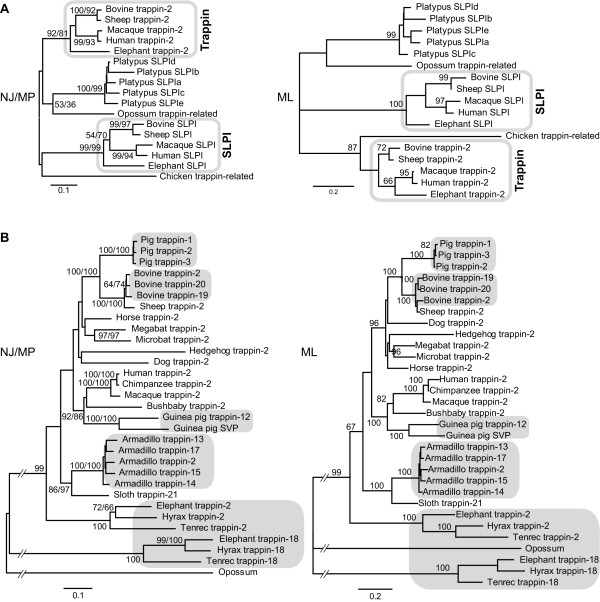
**Phylogenetic analyses of *trappin *genes**. (A) Phylogeny of *trappin*, *SLPI*, and *trappin*-related genes. The WAP-coding region of each gene was used for the analyses. (B) Phylogeny of *trappin *genes. Noncoding region of the *trappin *genes that was used for the analyses. The phylogenetic trees were constructed using neighbor joining (NJ), maximum parsimony (MP), and maximum likelihood (ML) methods. The NJ trees (left) with bootstrap values for both the NJ and MP methods and ML trees (right) are shown. *Trappin *genes with short nucleotide sequences were not included in the analyses, because inclusion of short sequences reduces the reliability of the analyses when we removed all the sites containing missing data and alignment gaps prior to the calculation (called the complete deletion in MEGA software). Bars indicate 10% replacement per site. Species- or lineage-specific *trappin *multigenes are shaded in gray.

Platypus *SLPI *genes show a stronger identity with *trappin-2 *(67%) than *SLPI *(55%) in the deduced amino acid sequences. The phylogenetic analysis for *trappin*, mammalian *SLPI*, platypus *SLPI*, and *trappin*-related genes of chicken and opossum is shown in Figure 2A. *Trappin*-related genes of chicken and opossum and platypus *SLPI *genes are not clearly categorized as *trappin *or *SLPI*.

### Identification of *trappin-2 *genes from various eutherian mammals

**Figure 3:**
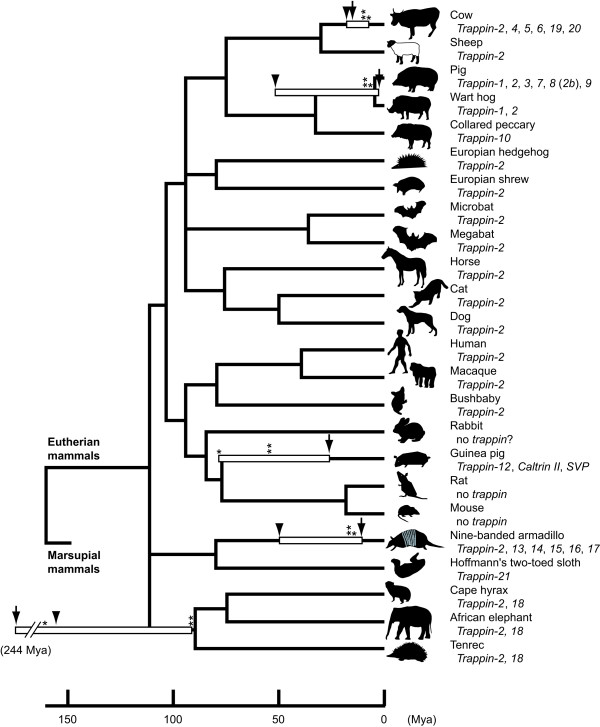
**History of the evolution of *trappin *genes in mammals**. Phylogeny of mammalian species with the time scale was generated based on the works by Kumar and Hedges [31], Hasegawa *et al*. [48], Nishihara *et al*. [24], Hallstrom and Janke [49], and Arnason *et al*. [50]. Ranges of the estimated dates for gene duplications are indicated by open boxes. Bayesian estimations of duplications using the nucleotide and the amino acid sequences of *trappin *genes are indicated by arrows and arrow heads, respectively. The dates of duplication estimated by MEGA software using single reference point are indicated by asterisks. The dates of duplications individually estimated by MEGA software for each species are indicated by double asterisks. Mya, million years ago.

We analyzed genome databases for various eutherian mammals including human, chimpanzee, rhesus macaque, bushbaby, mouse, rat, rabbit, dog, cat, cow, European shrew, European hedgehog, microbat, megabat, nine-banded armadillo, sloth, elephant, hyrax, and tenrec. Only mouse, rat, and rabbit lack *trappin *genes in their genome databases, but the other species have at least one *trappin *gene in their genome databases (Figure 3). All the newly identified *trappin *genes consist of three exons like previously analyzed *trappin *genes: exon 1 encodes a signal peptide, exon 2 encodes the TGS and WAP domains, and exon 3 encodes the 3' untranslated region (data not shown). We aligned the amino acid sequences of the WAP domains of those genes, and categorized them into two groups: the first group contained the catalytically important Met residue (asterisk in Figure 1C) and was named *trappin-2 *(Met rule); and the second group lacked the Met residue and was named according to the order of discovery. Most animals have a single *trappin-2 *gene (Figures 1C and 3). This finding suggests that the *trappin-2 *gene is the ancestral form of the *trappin *genes. According to this definition, the previously reported porcine trappin-8 should also be renamed *trappin-2b *as it also has the Met residue. Tenrec *trappin-18 *also has the Met residue at the catalytic site. However, the phylogenetic analyses using nucleotide sequences of the noncoding regions (introns, exon 3 and 5' and 3' flanking regions) clarified that this gene is closely related with *trappin-18 *genes of elephant and hyrax. Therefore we call this gene tenrec *trappin-18 *as an exception to the Met rule.

The average nonsynonymous and synonymous distances were calculated on 150 bp WAP-coding regions among the *trappin-2 *genes of the various species, *trappin *paralogs except for *trappin-2*, and all *trappin *genes (Table 1). In the WAP-coding region, the rate of nonsynonymous substitutions is lower than that of synonymous substitutions among *trappin-2*. The rates of nonsynonymous and synonymous substitutions are similar in the WAP-coding region among *trappin *paralogs except for *trappin-2*. These results suggest that the purifying selection is operating on the *trappin-2 *gene in various eutherian mammals.

**Table 1:** Purifying selection of *trappin-2 *genes.

	Trappin-2	Other trappin paralogs	All trappin genes
dn	0.190 ± 0.041	0.455 ± 0.092	0.369 ± 0.071

ds	0.333 ± 0.057	0.388 ± 0.066	0.377 ± 0.054

Dn/ds	0.57	1.17	0.98

Average non-synonymous and synonymous distances were calculated on 150 bp WAP-coding regions among *trappin-2 *genes of various species, the other *trappin *paralogs, and all *trappin *genes.

### Identification of novel *trappin *multigene families in armadillo and Afrotheria (elephant, tenrec, and hyrax)

Database analyses demonstrated the presence of six *trappin *genes in nine-banded armadillo (*Dasypus novemcinctus*), which were named *trappin-2 *and *trappins-13-17 *(Figures, 1 and 3). Afrotherian species such as the elephant (*Loxodonta africana*), tenrec (*Echinops telfairi*), and hyrax (*Procavia capensis*) had two *trappin *genes, which were named *trappin-2 *and *trappin-18 *(Figures, 1 and 3). We also found two novel *trappin *paralogs from the bovine genome database, and named them *trappin-19 *and *trappin-20 *(Figures 1D and 3).

Phylogenetic analyses of the noncoding regions of *trappin *genes from several mammalian species are shown in Figure 2B. All armadillo *trappins-13-17 *genes form a single branch with armadillo *trappin-2 *gene. Bovine *trappin-19 *and *trappin-20 *also share the same branch with bovine *trappin-2*. These results suggest that those genes are recently duplicated species specific paralogs. On the other hand, Afrotherian *trappin-18 *is divided near the root, suggesting that *trappin-18 *duplicated much earlier.

### Estimations of the dates for the duplication of *trappin *multigenes

A linearized tree was constructed by using the nucleotide sequences of *trappin *multigenes and the dates of the duplications were calculated with MEGA software. When the divergence time between Primate and Artiodactyla (96.2 Mya) was used as a reference point, the date of the duplications of *trappin *genes of pig, cow, armadillo, guinea pig, and Afrotheria (elephant, hyrax, and tenrec) were calculated as 7.0, 8.8, 15.9, 79.0, and 161 Mya, respectively (asterisks in Figure 3).

We next calculated the date of duplications individually for each species using the taxon pair that was most closely related to the node of interest as a reference point. We found that some of the trappin gene subfamilies were relatively young. For instance, when the divergence time between sheep and cow (18.3 Mya) was used as a calibration point, the date of the duplication events giving rise to the pig and bovine *trappin *gene families were calculated as 7.8 and 9.7 Mya, respectively. Similarly, when the divergence time between human and armadillo (96.2 Mya) was used as a reference point, the date of the duplication event giving rise to the armadillo *trappin *gene family was calculated as 15.5 Mya. On the other hand, certain trapping gene subfamilies appear to be more ancient. For example, when the divergence time between primate and rodent (61.7 Mya) was used as a reference point, the date the guinea pig *trappin *gene subfamily was estimated to have originated 55.2 Mya, and when the divergence time between elephant and tenrec (48.6 Mya) was used as a reference point, the Afrotherian *trappin *trappin gene subfamily was calculated to have originated 91.9 Mya (double asterisks in Figure 3).

We also estimated divergence times using the Bayesian method implemented in the BEAST software package [25]. The date of the duplications of *trappin *genes of pig, cow, armadillo, guinea pig, and Afrotheria were calculated as 3.3, 12.6, 11.4, 34.7, and 244 Mya (arrows in Figure 3), respectively, when the nucleotide sequences of *trappin *genes were used for the calculation. When amino acid sequences were used for the calculation, the date of the duplications of *trappin *genes of pig, cow, armadillo, and Afrotheria were calculated as 49.7, 18.3, 50.9, and 154 Mya (arrowheads in Figure 3), respectively. Thus, the dates calculated for nucleotide and amino acid sequence data are very different. The former are similar to the dates generated using the linearized tree method in pig, cow, and armadillo, and the latter are similar to the dates generated using the linearized tree method in Afrotheria. In pig, cow, and armadillo, the protein-coding regions of *trappin *multigenes have evolved rapidly (Table 2), and the dates calculated for amino acid sequence data showed larger values. In Afrotheria, since amino acid sequences evolve more slowly than nucleotide sequences and are, therefore, less prone to homoplasy over the long evolutionary time frames being considered, it is reasonable to suspect that the dates generated using nucleotide sequence data are not as reliable as those generated from acid sequence data for this particular study.

**Table 2:** Accelerated evolution of *trappin *multigenes.

Line	Region	Armadillo	Cow	Pig	Elephant	Hyrax	Guinea pig
A1	5' flanking region	0.059 ± 0.009 0.115 ± 0.042	0.090 ± 0.008 0.108 ± 0.013	0.010 ± 0.003 0.01 ± 0.003	0.610 ± 0.051 0.850 ± 0.108	0.935 ± 0.039 1.574 ± 0.136	ND

A2	exon 1 (signal peptide)	0.010 ± 0.007 0.016 ± 0.236	0.066 ± 0.026 0.156 ± 1.227	0.078 ± 0.026** 0.286 ± 47.78	0.433 ± 0.099 0.682 ± 0.744	0.708 ± 0.158 2.728 ± 3.455	0.318 ± 0.063 0.588 ± 0.242

A3	intron 1	0.060 ± 0.006 0.163 ± 0.033	0.101 ± 0.009 0.058 ± 0.011	0.017 ± 0.004 0.017 ± 0.004	0.914 ± 0.073 1.326 ± 0.188	0.807 ± 0.046 1.132 ± 0.098	0.397 ± 0.026 0.534 ± 0.034

A4	exon 2 (TGS and WAP)	0.235 ± 0.025** 0.442 ± 0.110	0.167 ± 0.019** 0.299 ± 0.067	0.220 ± 0.022** 0.307 ± 0.040	0.430 ± 0.049 0.577 ± 0.095	0.576 ± 0.066 0.953 ± 0.267	ND***

A5	intron 2	0.045 ± 0.010 0.131 ± 0.280	0.057 ± 0.011 0.094 ± 0.036	0.044 ± 0.008** 0.046 ± 0.008	0.750 ± 0.089 1.083 ± 0.240	0.645 ± 0.081 0.845 ± 0.135	0.403 ± 0.040 0.495 ± 0.066

A6	exon 3 (non coding)	0.077 ± 0.014 0.142 ± 0.095	0.026 ± 0.009 0.032 ± 0.018	0.004 ± 0.004 0.004 ± 0.005	0.420 ± 0.069 0.568 ± 0.161	0.662 ± 0.105 0.990 ± 0.238	0.417 ± 0.052 0.579 ± 0.113

A7	3' flanking region	0.026 ± 0.006 0.045 ± 0.022	0.038 ± 0.006 0.052 ± 0.014	0.016 ± 0.003 0.016 ± 0.003	0.615 ± 0.049 0.815 ± 0.100	0.862 ± 0.034 1.223 ± 0.075	0.363 ± 0.035 0.419 ± 0.050

B1	entire gene except coding region	0.054 ± 0.004 0.079 ± 0.008	0.053 ± 0.003 0.070 ± 0.006	0.019 ± 0.002 0.019 ± 0.002	0.716 ± 0.041 1.092 ± 0.082	1.540 ± 0.024 1.531 ± 0.054	0.376 ± 0.022 0.438 ± 0.032

C1	pre	0.012 ± 0.009 0.022 ± 297.0	0.064 ± 0.022 0.143 ± 1.791	0.071 ± 0.027** 0.097 ± 196000	0.271 ± 0.074 0.344 ± 0.130	0.591 ± 0.141 1.940 ± 5.811	0.318 ± 0.063 0.475 ± 0.671

C2	pre (non synonymous)	0.009 ± 0.009	0.043 ± 0.029	0.059 ± 0.037**	0.226 ± 0.079	0.489 ± 0.152	0.287 ± 0.060

C3	pre (synonymous)	0.020 ± 0.020	0.120 ± 0.058	0.068 ± 0.053**	0.384 ± 0.196	0.942 ± 0.530	0.405 ± 0.155

	dn/ds	0.5	0.4	0.9	0.6	0.5	0.7

D1	TGS	0.118 ± 0.023* 0.213 ± 0.145	0.195 ± 0.031** 0.387 ± 0.229	0.353 ± 0.039** 0.638 ± 0.137	0.604 ± 0.101 0.803 ± 0.214	0.977 ± 0.168 2.166 ± 1.289	0.474 ± 0.038 0.814 ± 0.056

D2	TGS (non synonymous)	0.147 ± 0.036*	0.211 ± 0.046**	0.355 ± 0.056**	0.724 ± 0.165	1.097 ± 0.322	0.452 ± 0.049

D3	TGS (synonymous)	0.057 ± 0.027	0.153 ± 0.056	0.288 ± 0.068**	0.355 ± 0.126	0.726 ± 0.203	0.533 ± 0.086

	dn/ds	2.6	1.4	1.2	2.0	1.5	0.8

E1	WAP	0.309 ± 0.040** 0.364 ± 2.671	0.145 ± 0.028** 0.325 ± 0.037	0.209 ± 0.038** 0.376 ± 0.084	0.337 ± 0.061 0.492 ± 0.171	0.439 ± 0.073 0.702 ± 0.284	0.601 ± 0.087 1.235 ± 1.552

E2	WAP (non synonymous)	0.326 ± 0.067**	0.163 ± 0.044**	0.248 ± 0.051**	0.310 ± 0.093	0.366 ± 0.096	0.609 ± 0.133

E3	WAP (synonymous)	0.315 ± 0.070**	0.091 ± 0.039	0.134 ± 0.060**	0.460 ± 0.152	0.632 ± 0.233	0.733 ± 0.249

	dn/ds	1.0	1.8	1.9	0.7	0.6	0.8

Average JC (upper) and TN (lower) distances of each region among the *trappin *multigenes for each species (A1--E3) and estimated times of gene duplication (F1--F2) are shown. For each species, the accelerated evolution of each region (A1--A7, C1--E3) was assessed by making comparisons against the average distances of non-coding regions (B2). The bold letters indicate the distances of regions which evolved faster than the non-coding regions of the same genes. * *P *< 0.05, ** *P *< 0.01. *** In guinea pig, there is only one trappin gene that has both the TGS and WAP domains. ND, no data; pre, signal peptide (pre-sequence).

### Synteny analyses around *trappin *genes

**Figure 4:**
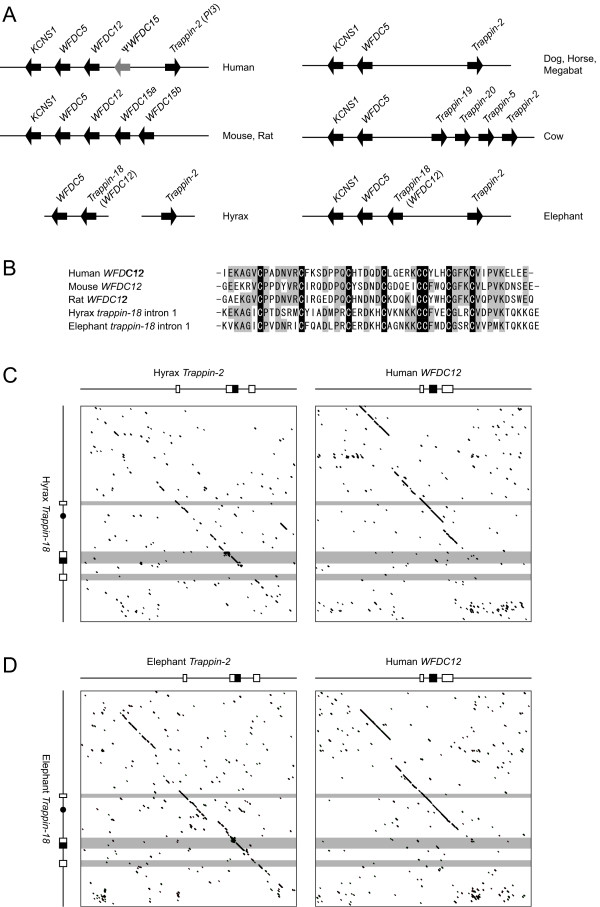
**Evolutional relationship between *trappin-18 *and *WFDC12***. (A) Schematic illustration of conserved synteny around *trappin*(s). Illustrations of human, mouse, rat, and dog loci were generated based on the works by Clauss *et al*. [20] and Hurle *et al*. [26]. Black and gray arrows indicate genes and pseudo genes, respectively. *KCNS*, potassium voltage-gated channel, member 1 (B) The WAP domains of *WFDC12 *and the WFDC12-like peptide encoded by intron 1 region of *trappin-18*. Conserved and semiconserved residues are indicated by light gray. Eight conserved Cys residues constituting the WAP motif signature sequence are shaded in black. (C) Harr plot analyses of the hyrax *trappin-18 *in comparison with the hyrax *trappin-2 *and human *WFDC12*. (D) Harr plot analyses of the elephant *trappin-18 *in comparison with the elephant *trappin-2 *and human *WFDC12*. Exons are indicated by boxes. WAP-coding regions are represented by black boxes. Black circles indicate regions encoding WFDC12 like peptide.

It has been demonstrated that the *trappin-2 *is mapped on the WAP four-disulphide core (WFDC) domain locus which contains a number of WFDC genes [17]. Moreover, the conserved synteny of WFDC loci has been studied for primates, rodents, and the dog [20,26]. To extend these studies, we analyzed the genes neighboring *trappin*s in species that were included in our study. Most scaffolds containing *trappin *genes were too short to analyze. However, we could analyze genes neighboring to horse *trappin-2*, megabat *trappin-2*, bovine *trappin*s, hyrax *trappin-18 *and elephant *trappin*s (Figure 4A). As reported previously for the dog WFDC locus, the horse, megabat, and bovine *trappin *genes flanked *WFDC5 *in opposed directions, whereas *WFDC12 *and *WFDC15 *were not found between *WFDC5 *and *trappin*(s). The bovine *trappin *genes were tandemly arrayed, suggesting that these genes arose by tandem gene duplication. In contrast, hyrax and elephant *trappin-18 *flanked *WFDC5 *in the same direction and was mapped to the same locus as *WFDC12*. Harr Plot analyses demonstrated that human *WFDC12 *is highly homologous to the 5'-flanking region, exon 1, and intron 1 of hyrax *trappin-18 *(Figure 4C). Similar homology was observed between human *WFDC12 *and elephant *trapin-18 *(Figure 4D).

Recently, Hurle *et al*. found that primate *trappin-2 *contains a pseudogene for *WFDC12 *in intron 1, and suggested that *trappin-2 *and *WFDC12 *have a common ancestral gene [26]. All *trappin *genes contained a pseudogene for *WFDC12 *in intron 1 (data not shown) except for Afrotherian *trappin-18*, which codes for a WFDC12-like peptide in intron 1 (Figure 4B).

### Accelerated evolution of TGS and WAP coding region *trappin *multigenes in armadillo, cow, and pig and positive selection of the WAP-coding region of pig *trappin *paralogs

The average distances of the 5'-flanking region, exon 1, intron 1, exon 2, intron 2, exon 3, and 3'-flanking region among *trappin *multigenes for each species were calculated (Table 2, line A1-A7). In armadillo *trappin*s, the average Jukes-Cantor (JC) distance between the exon 2 regions was 0.235 (Table 2, line A4), which is 4.4 times higher than that between the non-coding regions (0.054; Table 2, line B1). When we calculated the average Tamura-Nei (TN) distances with gamma correction, the value between exon 2 (0.442; Table 2, line A4) was also 5.6 times higher than that between the non-coding regions (0.079; Table 2, line B1). Fisher's exact test using the numbers of varied sites and common sites between the exon 2 regions (39 varied sites in 201 common sites) and those between the non coding regions (87 varied sites in 1691 common sites) demonstrated that the difference is significant (*P *< 0.01). A similar difference was not observed in the other regions.

In cow, the average distances between the exon 2 regions were 0.167 (JC method) and 0.299 (NJ method) (Table 2, line A4), and were 3.1 and 4.3 times, respectively, higher than those between the non-coding regions (Table 2, line B1; *P *< 0.01, Fisher's exact test). In pig, the average distances between the exon 2 regions (Table 2, line A4) were 12 and 16 times higher than those between the non-coding regions when calculated by the JC and TN methods, respectively (Table 2, line B1; *P *< 0.01). In contrast, there was no significant difference in the average distances between the exon 2 regions (Table 2, line A4) and those between the non-coding regions (Table 2, line B1) of elephant, hyrax, and guinea pig *trappin *genes. In pig, the average distances between the exon 1 regions and between the intron 2 regions of different genes (Table 2, lines A2 and A5) were also higher than those of the non-coding regions (Table 2, line B1) (*P *< 0.01).

Next, we calculated distance values for synonymous substitutions per site (ds) and non-synonymous substitutions per site (dn) for the signal peptide (pre peptide), TGS, and WAP coding regions (Table 2, line C1-E3), and compared against the average distance of the non-coding regions. In armadillo, dn of the TGS coding domain (*P *< 0.05) and both ds and dn of the WAP coding domain (*P *< 0.01) were significantly higher than the average distance of the non-coding regions. In cow, only dn of the TGS and WAP coding regions were significantly higher than the average distance of the non-coding regions (*P *< 0.01). In pig, both dn and ds of the signal peptide, TGS, and WAP coding regions were higher than the average distance of the non-coding regions.

To examine the presence of positive Darwinian selection, we compared dn and ds of the rapidly-evolved coding regions using Fisher's test. When the average values in each species were used for the analyses, we could not detect any statistically significant difference between dn and ds of all the *trappin *multigenes. However, the pairwise comparison matrix of dn and ds on the paralogs of each species demonstrated a dn/ds rate of 3.3 for porcine *trappin-2 *vs. *trappin-3*, 4.9 for porcine *trappin-2 *vs. *trappin-9*, and 5.2 for porcine *trappin-8 *vs. *trappin-9 *(*P *< 0.05) (Table 3). Because *trappin-2 *is the most conservative gene within the *trappin *family and porcine *trappin-8 *is the closest homolog of porcine *trappin-2*, these data indicate the positive Darwinian selection of porcine *trappin-3 *and *trappin-9*. Although the dn/ds rate between bovine *trappin-5 *and other bovine paralogs are as high as 2.8-5.8, the differences were not statistically significant (*P *= 0.07) (Table 3).

**Table 3:** Positive selection of species-specific *trappin *paralogs.

	pTr-2				
			
pTr-8	-	pTr-8			
		
pTr-1	1.5	1.3	pTr-1		
	
pTr-3	3.3*	3.8	3.0	pTr-3	
pTr-9	4.9*	5.2*	2.4	2.5	pTr-9

pTr-7	1.5	1.5	2.3	1.5	2.1

					
					
	bTr-2				
			
bTr-5	1.1	bTr-5			
		
bTr-19	1.2	3.9	bTr-19		
	
bTr-6	1.2	3.8	-	bTr-6	
	
bTr-20	2.6	5.8	3.0	1.3	
	
					
					
	aTr-2				
			
aTr-15	1.0	aTr-15			
		
aTr-16	0.7	0.7	aTr-16		
	
aTr-17	1.6	0.9	0.9	aTr-17	

aTr-13	1.4	1.3	1.4	1.4	aTr-13

aTr-14	1.9	1.8	1.7	1.6	1.6

Pairwise comparison matrixes of dn/ds rates are shown. p, pig; b, bovine; a, armadillo; Tr, trappin; * *P *< 0.05.

### Evaluation of the quality of genomic sequence with low coverage

The nucleotide substitutions between seven known cDNAs and corresponding exons in the genome databases were calculated and shown in Table 4. Some of these substitutions may have occurred as a result of sequencing errors or site-specific polymorphism within each species. Yet, we can still infer that the average rates of sequencing errors are lower than the substitution rates. In low coverage genomic sequences of armadillo, rabbit, cat, and elephant, the average substitution rates of the seven genes were 0.10-0.49%. In high coverage genomic sequences of cow and human, the average substitution rates of the seven genes were 0.23 and 0.06%, respectively. These estimates are not substantially different, suggesting that artifacts due to errors or polymorphism are negligible. In the case for armadillo *trappin *genes, for example, the 129-bp WAP-coding regions and the ~2.1-kb entire genes may contain less than 0.4 and 6-base sequence errors, respectively. Among armadillo *trappin *multigenes, the WAP-coding regions and the ~2.1-kb entire genes have 8-50 and 96-165-base substitutions, respectively. Thus, the sequencing errors of genomic sequences with low coverage appear to be negligible, although the caveat remains that we can not negate a small number of possible errors.

**Table 4:** Nucleotide substitutions between known cDNA and corresponding genomic sequences

	Armadillo (2 × coverage)	Rabbit (2 × coverage)	Cat (1.87 × coverage)	Elephant (2 × coverage)	Cow (7 × coverage)	Human (GRCh37)
*SDHA*	1/899	14/543	1/976	5/1222	ND	3/1650

*MDH2*	2/713	2/306	7/878	1/626	0/835	0/912

*ATP5B*	0/876	2/1237	4/1072	0/926	0/950	0/1335

*GAPDH*	6/413	ND	2/683	0/542	1/674	0/691

*SDHB*	0/476	1/468	0/586	ND	1/551	0/591

*CS*	0/646	0/672	2/564	2/677	2/653	0/673

*IDH1*	4/477	0/666	0/740	1/1077	7/1064	1/1114

total	13/4500	19/3892	16/5499	9/5070	11/4727	4/6966

substitution rate (%)	0.29	0.49	0.29	0.10	0.23	0.06

identity (%)	99.7	99.5	99.7	99.9	99.8	99.9

Numbers show the nucleotide substitutions per the length of the sequences used for the analysis. The scaffold, contig, or accession numbers of the sequences are shown in Supplementary Table S1 (additional file 1). ND, no data.

## Discussion

### Origin of *trappin *gene

Computer analyses of genome databases revealed that typical *trappin *is a eutherian mammalian specific gene. The typical *trappin *genes were found only in eutherian mammals and not other species including *Xenopus*, fish, sea squirt, insects, and *C. elegans*. The *trappin*-related genes were found in chicken and opossum. The computer analyses also showed that most eutherian mammalian species have a single *SLPI *gene, and platypus has multiple *SLPI *genes. The *trappin*-related genes of those animals and platypus *SLPI *genes show strong similarity with *trappin *in the WAP domain only, but all the other regions have no significant homology. Therefore, these genes may relate with the ancestoral WAP domain of *trappin*. Interestingly, platypus SLPI showed higher homology to the WAP domain of trappin-2 than that of mammalian SLPI. This strongly suggests that the WAP domain of trappin and SLPI share a common ancestor.

**Figure 5:**
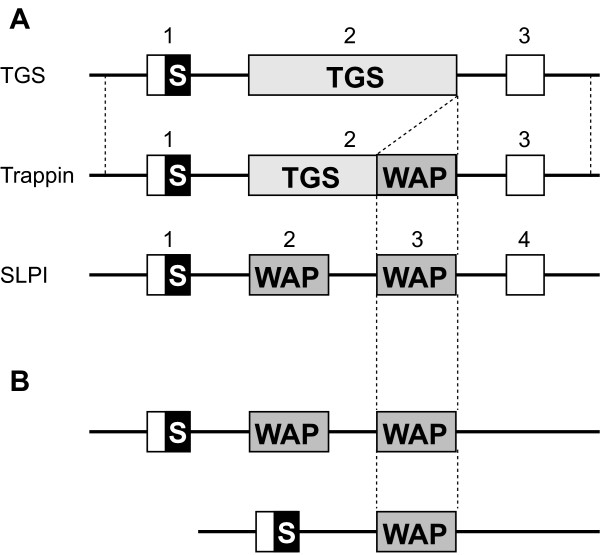
**Schematic representations of TGS, *trappin*, *SLPI*, and the *trappin*-related gene**. (A) The three-exon structure of mammalian TGS and *trappin *genes and the four-exon structure of mammalian SLPI gene are shown. (B) Structure of the *trappin*-related genes of chicken and opossum (upper) and platypus SLPI gene (lower). The homologous regions are shown by dotted lines. The exons are shown by boxes. TGS- and WAP-coding exons are indicated by light and dark gray, respectively. The signal-peptide-coding regions and noncoding regions are indicated by black and white boxes, respectively. S, signal-peptide-coding region.

Trappin is a protein that consists of TGS and WAP domains. SLPI is a two WAP-domain protein. The second WAP domain of SLPI and the WAP domain of trappin-2 are quite similar in their amino acid sequences and functions such as antiproteolytic and antimicrobial activities, suggesting an ancestral relationship. However, except for the WAP-coding regions, there is no significant homology in the nucleotide sequences between *trappin *and *SLPI*. Interestingly, *trappin *genes are known to have weak but significant similarity with other TGS genes in introns, TGS-coding, and noncoding regions (Figure 5) [27]. This mosaic pattern of homology in *trappin *genes indicates that *trappin *genes originated from the TGS gene and obtained a WAP domain possibly by exon-shuffling. Afrotherian *trappin-18 *codes for a WFDC12-like peptide in intron 1 region, and the other *trappin*s contain a pseudogene for *WFDC12 *in intron 1. These results support the hypothesis of Hurle *et al*. [26] that *trappin *and *WFDC12 *are derived from a common ancestral gene which codes for both trappin and WFDC12.

### Evolution of *trappin *genes in eutherian mammals

Nineteen species of eutherian mammals were analyzed by a search for the presence of *trappin *genes within their genome databases, and the results were combined with those of previous experimental analyses of human [17,23], pig [18,19], wart hog [19], collared peccary [19], cow [28], sheep [16], and guinea pig [21,22]. In total, we could compare the *trappin *genes from 24 eutherian mammals (Figure 3). Within the 24 species analyzed, we could isolate *trappin *genes from 21 species. A single *trappin-2 *gene was found in 11 species, and multiple *trappin *genes were found in 8 species. These results indicate that *trappin-2 *is the most common and is an ancestral form while the other *trappins *are specie-specific paralogs. We could not find *trappin *genes in three mammalian species: mouse, rat, and rabbit. Our experimental analyses (data not shown) and the integrity of the genome databases of mouse and rat suggest that mouse and rat lack *trappin *genes in their genome [20]. In mouse and rat, other WAP-motif containing proteins such as SLPI and SWAMs may compensate the function of trappin. In the case of rabbit, it is not certain whether rabbit really lacks *trappin *genes or rabbit has a *trappin *gene that has not yet been analyzed by the genome project.

By computer analyses of genome databases, we found that the nine-banded armadillo as well as pig and cow also have recently-duplicated *trappin *multigene. The computer analyses of bovine genome databases also revealed two novel *trappin *paralogs and the sequences of the introns and flanking regions, which enabled the detailed evolutional analyses of bovine *trappin *multigenes. As previously reported porcine *trappin *multigenes, the WAP-coding regions of the *trappin *multigenes of armadillo and cow were shown to have evolved under accelerated evolution. Only dn was accelerated in the WAP coding regions of bovine *trappin*s, and both dn and ds of the WAP coding regions were accelerated in armadillo and porcine *trappin*s. The accelerated substitutions of non-synonymous sites of WAP-coding regions may be explained by positive Darwinian selection or relaxation of functional constraints, because we observed statistically significant positive selection of the WAP coding regions of porcine *trappin-3 *and *trappin-9 *but no significant difference between dn and ds of other *trappin*s (Table 3). However, the question why synonymous substitutions are also accelerated can not be interpreted simply by the existence of positive Darwinian selection or relaxation of functional constraint. The mechanism whereby the synonymous substitutions are accelerated must be clarified by future studies.

The molecular clock and Bayesian analyses using the nucleotide sequences estimated the date of duplication as 11.4-15.9, 8.8-12.6, and 3.3-7.8 Mya for *trappin *multigenes of armadillo, cow, and pig, respectively. These results are consistent with previous experimental analyses demonstrating that the collared peccary that was separated from porcine 33 Mya [29,30], and sheep, which was separated from bovine 19.6 Mya [31], do not have *trappin *multigenes [16,19]. The findings of recently-duplicated accelerated-evolved *trappin *multigenes in three individual species demonstrate that mammalian genomes have the potential to form *trappin *multigenes in several million years. The selective pressure that formed the *trappin *multigenes may relate with some pathogens, and the variety of amino-acid sequences in the WAP-domain may contribute to the acquisition of antimicrobial activities for a large spectrum of pathogens. Tissue distribution of *trappin *paralogs in pig and cow has been shown to vary among genes: porcine *trappin-2 *is expressed in the trachea and the large intestine, porcine *trappin-1 *in the small intestine, bovine *trappin-2 *in the epidermis and the tongue, bovine *trappin-4 *in the trachea and the tongue, and bovine *trappin-5 *in the trachea [28]. Therefore, the selective pressures might also affect the regulation of the tissue-specific expression of *trappin *genes.

Our previous analyses revealed that guinea pig has a *trappin-12 *gene [22] and two derivative genes, *SVP *[32] and *caltrin II *[19]. *SVP *and *caltrin II *genes have significant homology with *trappin *including introns, noncoding region of exons, and flanking regions, but lack WAP and TGS domains, respectively. The molecular clock analysis estimated the date of the duplication of the guinea pig genes as 34.7-79.0 Mya. This date of duplication is much earlier than those of pig, cow, and armadillo.

In Afrotherians we found two *trappin *genes, *trappin-2 *and *trappin-18*, whose date of duplication was estimated as 91.9-244 Mya. This date is surprising, because it is earlier than the date of the periods of divergence of the major orders of eutherian mammals (70-10 Mya) [24,31], and suggests that the duplication of *trappin-18 *occurred in the ancestors of the eutherian mammals before the divergence of the species. In this context, most species lack *trappin-18*, however, only Afrotheria has retained the gene. The reason is still unknown, but it is conceivable that trappin-18 increases resistance to Afrotheria-specific pathogen. Another possible alternative explanation is that *trappin-18 *underwent substitutions at a faster rate per year than other *trappin *genes and that lead to the duplication time being overestimated.

## Conclusions

• Typical *trappin *genes are only found in the genome sequences of eutherians but not in those of other vertebrate species.

• *Trappin-2 *is the most widely distributed and is the strongest candidate of the ancestral forms of *trappin*. Recently-duplicated species-specific *trappin *paralogs are present in the genomes of armadillo, pig, and cow, and the non-synonymous sites of those genes have undergone accelerated evolution as a result of positive Darwinian selection or relaxation of functional constraint.

• Synonymous sites of recently-duplicated *trappin *paralogs of armadillo and pig have also undergone accelerated evolution by unknown mechanisms.

• The anciently-duplicated *trappin-18 *gene is only retained in afrotherian species and is a fossil molecule of the *trappin *gene family.

## Methods

### Isolation of *trappin *genes from various animal species

The genome database of various species (URL: http://www.ensembl.org/index.html) [33] were screened using the amino-acid sequence of human trappin-2. The exon-intron organization was estimated by comparing it with that of the human *trappin-2 *gene. The nucleotide sequences of the *trappin *genes were deposited in the DDBJ/EMBL/GenBank DNA databases as third party annotations (TPAs) under accession numbers BR000322 to BR000327 and BR000708 to BR000720.

### Evaluation of the quality of genomic sequence with low coverage

To evaluate the quality of genomic sequences with low coverage, we compared known cDNA sequences with those of corresponding exons in the genome databases. We used cDNA sequences for *SDHA*, *MDH2*, *ATP5B*, *GAPDH*, *SDHB*, *CS*, and *IDH1 *which was determined by Kullberg *et al*. [34]. The corresponding exons of armadillo (2 × coverage), rabbit (2 ×), cat (1.87 ×), elephant (2 ×), cow (7 ×), and human (Genome Reference Consortium GRCh37 assembly) were isolated and the numbers of nucleotide substitutions between the sequences of cDNA and the genome databases were calculated for each species using MEGA software [35]. The sequences used for the analysis are shown in Supplemental Table S1 (see Additional file 1).

### Phylogenetic analyses

Nucleotide sequences of the WAP-coding regions of *trappin*, *SLPI*, and *trappin*-related genes were used to analyze their phylogenetical relationship. The introns, exon 3 (noncoding exon), and 3'-noncoding regions were used to analyze recent evolution of *trappin *genes in eutherian mammals. The nucleotide sequences were aligned using ClustalW software [36], and the best fit/gap placement was confirmed manually. Phylogenetic analysis was performed by the neighbor-joining (NJ) method [37,38] and maximum parsimony (MP) method [38] with 2,000 bootstrap replicates using MEGA software [35] or the maximum likelihood (ML) method with 200 bootstrap replicates using PHYML [39] plugin for Geneious software http://www.geneious.com. The sequences used are as follows with the accession numbers in parentheses: human (*Homo sapiens*) *trappin-2 *(D13156) and *SLPI *(X04502); chimpanzee (*Pan troglodytes*) *trappin-2 *(XM_514671) and *SLPI *(DP000037); macaque (*Macaca mulatta*) *trappin-2 *(XM_00110935) and *SLPI *(DP000043); bushbaby (*Otolemur garnettii*)*trappin-2 *(BR000708) and *SLPI *(DP000040); mouse (*Mus musculus*) *SLPI *(AF002719); rat (*Rattus norvegicus*) *SLPI *(AAHX01026351); guinea pig (*Cavia porcellus*) *trappin-12 *(AB161363), *caltrin II *(AB161364) and *SVP *(U59711); European shrew (*Sorex araneus*) *trappin-2 *(BR000713) and *SLPI *(AALT01303048); European hedgehog (*Erinaceus europaeus*) *trappin-2 *(BR000714) and *SLPI *(AANN01307740); microbat (*Myotis lucifugus*) *trappin-2 *(BR000712) and *SLPI *(AAPE01410948); megabat (*Pteropus vampyrus*) *trappin-2 *(ABRP01168531) and *SLPI *(ABRP01290205); horse (*Equus caballus*) *trappin-2 *(XM_001503186) and *SLPI *(XP_001503242); dog (*Canis familiaris*) *trappin-2 *(BR000710) and *SLPI *(AAEX02024101); cat (*Felis catus*) *trappin-2 *(BR000711) and *SLPI *(AANG01238466); bovine (*Bos taurus*) *trappin-2 *(AJ223216), *trappin-4 *(AJ223217), *trappin-5 *(AJ233218), *trappin-6 *(AB011010), *trappin-19 *(BR000718), *trappin-20 *(BR000719) and *SLPI *(AAFC03003522); sheep (*Ovis aries*) *trappin-2 *(NM_001035224) and *SLPI *(AY346135); porcine (Sus scrofa) *trappin-1 *(D50320), *trappin-2 *(D50319), *trappin-3 *(D50321), *trappin-7 *(D50323), *trappin-8 *(D50322), *trappin-9 *(AB003285) and *SLPI *(NM_213870); elephant (*Loxodonta africana*)*trappin-2 *(BR000716), *trappin-18 *(BR000717) and *SLPI *(AAGU01360578); hyrax (*Procavia capensis*) *trappin-2 *(ABRQ01439157), *trappin-18 *(ABRQ01336046), and *SLPI *(ABRQ01352342); tenrec (*Echinops telfairi*) *trappin-2 *(BR000715), *trappin-18 *(AAIY01696839), and *SLPI *(AAIY01696839); wart hog (*Phacochoerus aethiopicus*) *trappin-1 *(AB003282) and *trappin-2 *(AB003281); collared peccary (*Pecari tajacu*) *trappin-10 *(AB003283); hippopotamus (*Hippopotamus amphibius*) *trappin-11 *(AB003284); nine-banded armadillo (*Dasypus novemcinctus*) *trappin-2 *(BR000322), *trappin-13 *(BR000323), *trappin-14 *(BR000324), *trappin-15 *(BR000325), *trappin-16 *(BR000326), *trappin-17 *(BR000327) and *SLPI*; sloth (*Choloepus hoffmanni*) *trappin-21 *(ABVD01210669) and *SLPI *(ABVD01323747); platypus (*Ornithorhynchus anatinus*) *SLPIa *(AAPN01348542), *SLPIb *(AAPN01336636), *SLPIc *(AAPN01050486), *SLPId *(AAPN01048517) and *SLPIe *(AAPN01030446); chicken (*Gallus gallus*) *trappin*-related protein (NC_006107); finch (*Taeniopygia guttata*) *trappin*-related protein (ABQF01028586); and opossum (*Monodelphis domestica*) *trappin*-related protein (BR000720).

### Molecular clock analysis and Bayesian divergence time estimation

The introns, exon 3 (noncoding exon), and 5'- and 3'-noncoding regions of pig, cow, armadillo, guinea pig, and Afrotheria (elephant and hyrax) *trappin*s were aligned, and a phylogenetic tree was constructed by the NJ method. A linearized tree was constructed and the dates of the duplication events of *trappin *genes were calculated by MEGA 3.1 using the divergence time between Primate and Artiodactyla (96.2 Mya) [40] as a calibration point for dating.

As an additional method to investigate divergence times, we used the Bayesian method implemented in the software package BEAST 1.4.8 [25]. To generate divergence times, the following nine fossil calibration points were taken from the work by Benton and Donoghue [40] and implemented as priors in the analysis of both DNA sequence and amino acid sequence data: (1) human-chimp: 6.5 Mya; (2) human-Macaque: 23.5 ± 0.5 Mya; (3) dog-cat: 43 ± 0.2 Mya; (4) cow-sheep: 18.3 ± 0.1 Mya; (5) cow-dog 96.2 ± 0.9 Mya; (6) human-cow: 96.2 ± 0.9 Mya; (7) human-armadillo: 96.2 ± 0.9 Mya; (8) tenrec-elephant: 48.6 ± 0.2 Mya; (9) human-opossum: 124.6 ± 0.1 Mya. The chains were run until convergence was reached (i.e., until the effective sample size for each parameter exceeded 200), which was 93 million states for the DNA sequence data and 10 million states for the amino acid sequence data. The HKY + gamma model was used for the analysis of the DNA sequence data, and the WAG model was used for the analysis of the amino acid sequence data. For both sequence data types, the birth-death speciation process was used as a tree prior.

### Calculation of nucleotide substitution rates

Nucleotide sequences were separated into the following regions: the 5'-flanking region, exon 1, intron 1, exon 2, the WAP-coding region of exon 2, intron 2, exon 3, and the 3'-flanking region. These regions were aligned separately using ClustalW software. Jukes-Cantor (JC) distances [41] and Tamura-Nei (TN) distances [42] were calculated using MEGA software [35]. For the calculation of TN distances, we estimated the gamma shape parameter using MrBayes [43] plugin for Geneious software. Distance values for the synonymous substitutions per site (ds) and non-synonymous substitutions per site (dn) of the signal-peptide-coding region of exon 1, TGS- and WAP-coding regions of exon 2 were calculated using the modified Nei-Gojobori (NG) method [44]. Standard errors were computed using the bootstrap method [45] with 2,000 replicates. Fisher's exact test was used for the statistical analyses [46].

### Synteny and Harr plot analyses

Synteny of neighboring genes of the *trappin *genes was investigated by surveying neighboring genes on horse genome cont2.26764 (AAWR02026765), megabat genome cont1.168530 (ABRP01168531), cow chromosome 13 (DAAA02036736) [47], hyrax genome cont1.336045 (ABRQ01336046), elephant SuperContig scaffold_19, human chromosome 20, mouse chromosome 2, rat chromosome 3, and dog chromosome 24. Harr plot analyses were performed at a 23/40 nucleotide stringency using Genetyx-win software (Genetyx Co., Tokyo).

## List of abbreviations

TGS: transglutaminase substrate; WAP: whey acidic protein; SVP: seminal vesicle clotting protein; SLPI: secretory leukocyte proteinase inhibitor; Mya: million years ago; ds: distance values for synonymous substitutions per site; dn: distance values for non-synonymous substitutions per site; NJ: neighbor-joining; MP: maximum parsimony; JC: Jukes-Cantor; NG: Nei-Gojobori.

## Authors' contributions

AK and SH planned and designed the study. AK, YF, and AR performed the analyses. AK, AR, and SH wrote the manuscript. All authors read and approved the final manuscript.

## Supplementary Material

Additional file 1 **Nucleotide sequences used for the evaluation of the quality of genomic sequence with low coverage**. Accession numbers for the cDNAs (upper) and scaffold, contig, or accession numbers of the genomic sequences (lower) are shown. ND, no data. Click here for file
