# Supplementary material for: Evolution of trappin genes in mammals
Source: BMC Evol Biol. 2010 Jan 29;10:31. doi: 10.1186/1471-2148-10-31 (PMC2831891; doi:10.1186/1471-2148-10-31)
Supplement: Additional file 1 — Nucleotide sequences used for the evaluation of the quality of genomic sequence with low coverage. Accession numbers for the cDNAs (upper) and scaffold, contig, or accession numbers of the genomic sequences (lower) are shown. ND, no data. [file 1471-2148-10-31-S1.DOCX]

**Supplemental Table 1: Nucleotide sequences used for the evaluation of the quality of genomic sequence with low coverage**

|  | Armadillo  (2 x coverage) | Rabbit  (2 x coverage) | Cat  (1.87 x coverage) | Elephant  (2 x coverage) | Cow  (7 x coverage) | Human  (GRCh37) |
| --- | --- | --- | --- | --- | --- | --- |
| *SDHA* | DQ402973  Genescaffold_20760 | DQ402980  GeneScaffold_6147 | DQ402986  c524202688.Contig1 | DQ402972  GeneScaffold_6060 | DQ402990  ND | DQ402982  NT_006576 |
| *MDH2* | DQ402948  GeneScaffold_2722 | DQ402954  GeneScaffold_3736 | DQ402961  c416801229.Contig1 | DQ402947  GeneScaffold_2555 | DQ402965  NW_001494318 | DQ402957  NT_007933 |
| *ATP5B* | DQ403098  GeneScaffold_6054 | DQ403105  GeneScaffold_610 | DQ403111  c468001642.Contig1 | DQ403097  cont3.7417 | DQ403115  NW_001495046 | DQ403107  NT_029419 |
| *GAPDH* | DQ403048  scaffold_35003 | DQ403051  ND | DQ403036  c421000969.Contig1 | DQ403047  GeneScaffold_3317 | DQ403066  NW_001494990 | DQ403057  NT_009759 |
| *SDHB* | DQ402998  GeneScaffold_2186 | DQ403005  GeneScaffold_2028 | DQ403011  c435900925.Contig2 | DQ402997  ND | DQ403015  NW_001494707 | DQ403007  NT_004610 |
| *CS* | DQ403123  GeneScaffold_3565 | DQ403130  GeneScaffold_3282 | DQ403136  c498002162.Contig1 | DQ403122  GeneScaffold_3344 | DQ403140  NW_001495046 | DQ403132  NT_029419 |
| *IDH1* | DQ403073  GeneScaffold_6272 | DQ403080  GeneScaffold_3190 | DQ403085  c440301251.Contig1b10 | DQ403072  GeneScaffold_6217 | DQ403090  NW_001494667 | DQ403082  NT_005403 |

Accession numbers for the cDNAs (upper) and scaffold, contig, or accession numbers of the genomic sequences (lower) are shown. ND, no data.
